# Epithelium-Specific ETS (ESE)-1 upregulated GP73 expression in hepatocellular carcinoma cells

**DOI:** 10.1186/2045-3701-4-76

**Published:** 2014-12-08

**Authors:** Fang Wang, Qi Long, Yu Gong, Longbo Hu, Hong Zhang, Peter Oettgen, Tao Peng

**Affiliations:** State Key Laboratory of Respiratory Disease, Guangzhou Institutes of Biomedicine and Health, Chinese Academy of Sciences, Guangzhou, 510530 China; Guangzhou Hoffmann Institute of Immunology, College of Basic Sciences, Guangzhou Medical University, Guangzhou, 510182 China; Guangzhou Overseas Chinese Hospital, Guangzhou, 510630 China; Division of Immunology, Beth Israel Deaconess Medical Center and Harvard Medical School, Boston, Massachusetts 02215 USA; Max Planck Institute for Molecular Biomedicine, Röntgenstraße 20, 48149 Münster, Germany

**Keywords:** GP73, GOLPH2, GOLM1, ESE-1, Liver inflammation, HCC

## Abstract

**Background:**

Golgi protein-73 (GP73) is a Golgi transmembrane glycoprotein elevated in numerous liver diseases. Clinically, GP73 is strongly elevated in the serum of HCC patients and is thus regarded as a novel potential biomarker for HCC. However, the mechanism leading to GP73 dysregulation in liver diseases remains unknown.

**Results:**

This study determined that epithelium-specific ETS (ESE)-1, an epithelium-specific transcription factor, and GP73 expressions were induced by IL-1β stimulation *in vitro*, and both were triggered during liver inflammation *in vivo*. In hepatocellular carcinoma cells, the overexpression of ESE-1 induced GP73 expression, whereas its knock-down did the opposite. Mechanistically, ESE-1 activated GP73 expression by directly binding to its promoter.

**Conclusions:**

Our findings supported a novel paradigm for ESE-1 as a transcriptional mediator of GP73. This study provided a possible mechanism for GP73 upregulation in liver diseases.

**Electronic supplementary material:**

The online version of this article (doi:10.1186/2045-3701-4-76) contains supplementary material, which is available to authorized users.

## Background

Golgi protein-73 (GP73), also known as GOLPH2 or GOLM1, is a type II Golgi transmembrane glycoprotein [1] and is predominantly expressed in epithelial cells [2]. It is upregulated in numerous liver diseases, including virus-induced hepatitis (e.g., hepatitis B and C virus), alcohol-induced liver disease, autoimmune hepatitis [3, 4], liver cirrhosis [4], and hepatocellular carcinoma cells (HCC) [5–7]. Clinically, GP73 is strongly elevated in the serum of HCC patients [8] and is thus regarded as a novel potential biomarker for HCC [5–7].

Previous studies showed that GP73 is induced under inflammatory conditions [3, 9–11]. In HepG2 and Hep3B cells, GP73 expression is elevated after treatment with proinflammatory cytokine IL-6 [9]. Increased GP73 expression in SK-Hep-1 cells is associated with interferon gamma (IFN-γ) stimulation [3]. *In vivo*, GP73 is upregulated in a mouse model of CCl_4_-induced cirrhosis [10]. However, the mechanism by which these extracellular signals trigger GP73 expression remains unclear.

ESE-1, also known as ELF-3, is a member of ESE subfamily of ETS transcription factors. It is exclusively expressed in epithelial cells and mainly contains two putative DNA binding domains, namely, the ETS and A/T hook domains [12]. Its ETS domain commonly binds a core consensus sequence of GGAA [13]. Similar to GP73, ESE-1 is also induced by proinflammatory factors such as IL-1β, tumor necrosis factor-α [14], and lipopolysaccharide (LPS) [15, 16]. Moreover, proinflammatory cytokine IL-1β induces ESE-1 expression in chondrocytes [17] and prostate cancer cells [18].

In this study, we demonstrated that similar to ESE-1, GP73 expression was also induced upon IL-1β stimulation, and was upregulated by ESE-1 in HCC cells. Mechanistically, we identified that ESE-1 activated GP73 expression by directly binding to its promoter. Thus, ESE-1 was a novel transcriptional regulator of GP73 in liver diseases.

## Results

### IL-1β stimulated both ESE-1 and GP73 expressions

**Figure 1:**
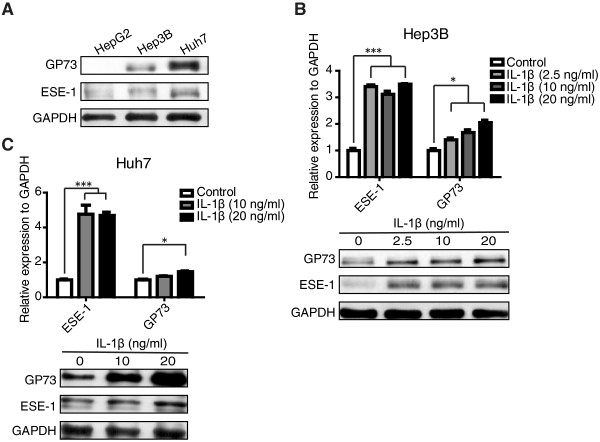
**ESE-1 and GP73 expressions were induced by IL-1β stimulation** ***in vitro*** **. (A)** The basal levels of ESE-1 and GP73 expressions in HepG2, Hep3B, and Huh7 cells were analyzed by conducting Western blot. **(B)** Hep3B cells were stimulated with different doses of IL-1β (2.5, 10, and 20 ng/mL) for 48 h. IL-1β-induced ESE-1 and GP73 mRNA and protein levels were analyzed by performing qPCR and Western blot. **(C)** Huh7 cells were stimulated with different doses of IL-1β (10 and 20 ng/mL) for 48 h. ESE-1 and GP73 mRNA and protein levels were analyzed with qPCR and Western blot. The values were normalized to *GAPDH*. Western blot was reprobed for GAPDH as a loading reference control.

Previous studies reported that under inflammatory conditions, GP73 expression is elevated in hepatocytes [3, 9]. Since ESE-1, an epithelial specific transcription factor, is also induced by proinflammatory cytokine IL-1β [17, 18], we asked whether ESE-1 played a role in the regulation of GP73 expression. By first comparing the basal levels of ESE-1 and GP73 proteins in different HCC cell lines (HepG2, Hep3B, Huh7), we found that Huh7 cells with high level of ESE-1 protein exhibited high level of GP73, whereas HepG2 cells with minimal ESE-1 protein showed minimal GP73 (Figure 1A). Similar correlation of ESE-1 and GP73 expression was also observed in other human and mouse hepatocytes (Additional file 1: Figure S1). To determine whether the expression of GP73 is also stimulated by IL-1β, Hep3B and Huh7 cells were subsequently treated with different doses of IL-1β. Both qPCR and Western blot analyses indicated that compared with the untreated control in Hep3B (Figure 1B) and Huh7 (Figure 1C) cells, ESE-1 was increased after IL-1β treatment, and that GP73 was also increased in a dose-dependent manner. These results indicated that ESE-1 and GP73 were induced in response to IL-1β stimuli.

**Figure 2:**
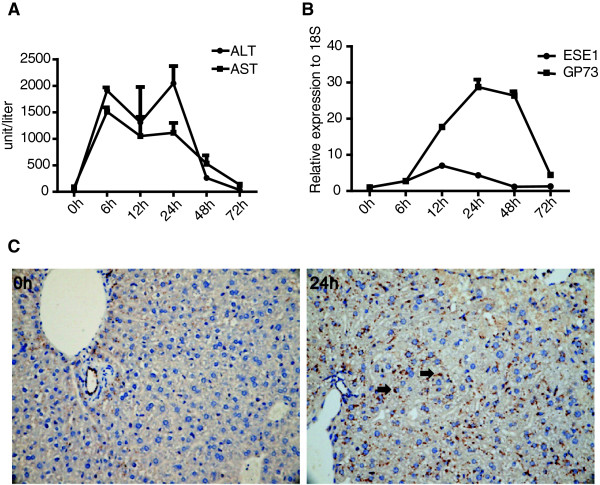
**ESE-1 and GP73 expressions were triggered during liver inflammation** ***in vivo*** **.** The mouse liver inflammation model was constructed by intraperitoneally injecting LPS (35 ng/kg) and D-galactosamine (250 mg/kg). **(A)** The levels of ALT and AST in mice sera were measured by full automatic blood analyzer at different time points during liver inflammation. **(B)** The mRNA levels of mouse ESE-1 and GP73 in liver tissues were measured with qPCR. **(C)** Mouse GP73 expression levels in liver tissues were analyzed at 0 and 24 h by accomplishing immunohistochemistry. Black arrows depicted the location of GP73 protein.

To determine ESE-1 and GP73 expressions under proinflammatory conditions *in vivo*, a mouse liver inflammation model was used. Mice were intraperitoneally injected with LPS and D-galactosamine [19]. Mice sera were collected at different time points, and the levels of alanine aminotransferase (ALT) and aspartate aminotransferase (AST) were measured. The levels of ALT and AST, as the criteria for inflammation response, peaked at 6 h post injection, suggesting that liver inflammation was induced. Inflammation was recovered after 72 h, as indicated by the decreased ALT and AST elevation (Figure 2A). The liver tissues of the mice were collected at different time points, and the mRNA levels of ESE-1 and GP73 were analyzed using qPCR. ESE-1 expression peaked at 12 h, whereas GP73 expression peaked at 24 h. Both expressions finally recovered to normal level within 72 h (Figure 2B). The results of immunohistochemical analysis verified that GP73 expression was induced by liver inflammation (Figure 2C).

**Figure 3:**
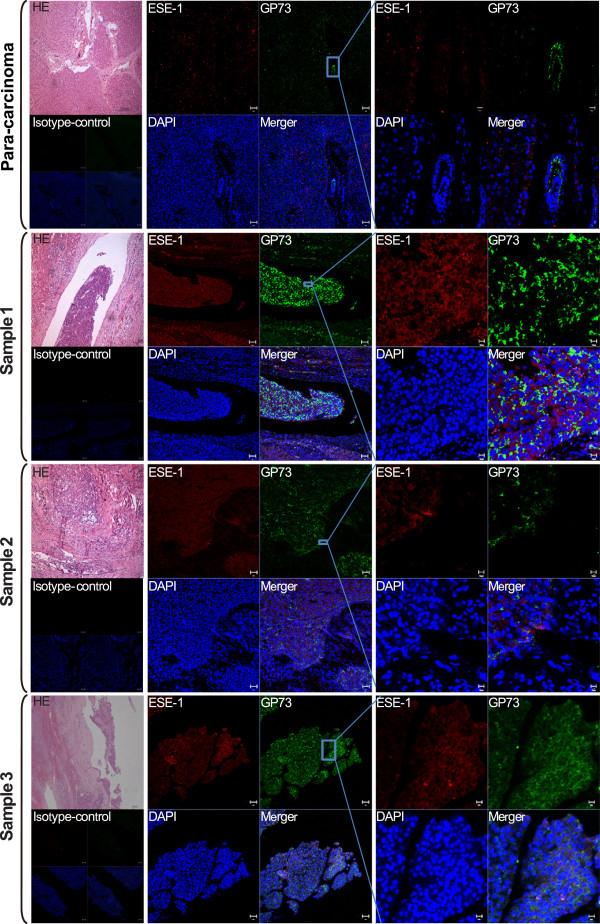
**ESE-1 and GP73 were elevated in the liver tissues of patients with HCC.** Liver tissues of HCC patients (*n* = 5) were collected and fixed by formaldehyde. Hematoxylin–eosin (HE) staining was used to present the pathological changes in liver tissues. ESE-1 (red fluorescence) and GP73 (green fluorescence) expression levels were analyzed by conducting immunofluorescence. Nuclei (blue fluorescence) were counterstained with DAPI. The isotype-matched IgG was used as a negative control.

Next, we determined ESE-1 and GP73 expressions in human HCC tissue samples using immunofluorescence with serial sections of samples. HE staining was used to present the pathological changes in liver tissues. Consistent with the previous reports, GP73 was located in the Golgi body [20], whereas ESE-1 was distributed in the cytoplasm [21, 22]. The regions with higher expression level of ESE-1 also showed higher GP73 expression, whereas in the para-carcinoma tissue, both proteins displayed lower expressions. The isotype-matched control samples exhibited no staining (Figure 3).

These studies collectively identified that ESE-1 and GP73 expressions were induced in response to IL-1β stimuli *in vitro* and were triggered during liver inflammation *in vivo*. Both expressions were elevated in HCC patient samples.

### ESE-1 upregulated GP73 expression in HCC cells

**Figure 4:**
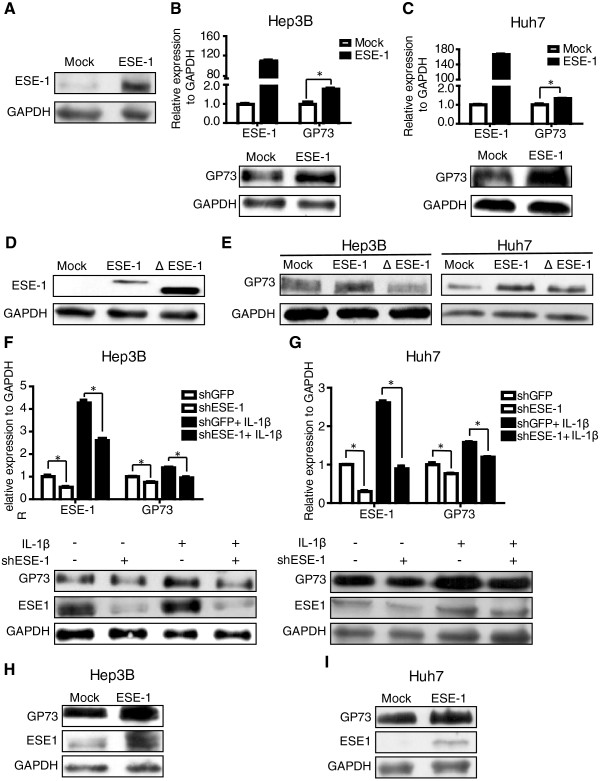
**ESE-1 upregulated GP73 expression in HCC cells. (A)** pCR3.1-ESE-1 plasmid was transfected into HEK293T cells and ESE-1 expression was tested by Western blot 36 h after transfection. **(B)** pCR3.1-ESE-1 plasmid was transfected into Hep3B cells for 36 h, and GP73 mRNA and protein levels were analyzed with qPCR and Western blot, respectively. **(C)** GP73 mRNA and protein levels were analyzed by adopting qPCR and Western blot in Huh7 cells transfected with pCR3.1-ESE-1 plasmid. **(D)** pCR3.1-ΔESE-1 plasmid with deletion ETS domain of ESE-1 was constructed and transfected into HEK293T cells. ΔESE-1 expression was confirmed by Western blot. **(E)** The empty vector (Mock) or ESE-1 or ΔESE-1 was transfected into Hep3B and Huh7 cells, and GP73 expression was tested by Western blot. **(F)** ESE-1 was downregulated in Hep3B and **(G)** Huh7 cells by infecting them with virus carrying the shRNA expression cassette against ESE-1 (shESE-1) or a non-target control (shGFP) with or without IL-1β stimulation. After 96 h, the mRNA and protein levels of ESE-1 and GP73 were analyzed with qPCR and Western blot, respectively. **(H)** Hep3B and **(I)** Huh7 cells infected with shESE-1 virus particles were transfected with pCR3.1-ESE-1 plasmid at 48 h post-infection. At 48 h after transfection, cells were harvested, and ESE-1 and GP73 proteins were analyzed with Western blot.

As previously indicated, ESE-1 and GP73 expressions were induced by IL-1β stimulation *in vitro*. We wondered whether ESE-1 regulated GP73 expression. An ESE-1 expression plasmid was constructed, and its expression was confirmed by transfecting pCR3.1-ESE-1 plasmid into 293T cells (Figure 4A). When ESE-1 was overexpressed in Hep3B cells, the transcript and protein levels of GP73 increased accordingly (Figure 4B). Similar results were also confirmed in Huh7 cells (Figure 4C).

To further confirm that ESE-1 is responsible for the elevated GP73 expression, a mutant form of ESE-1 [12] (lacking ETS domain, which was required for ESE-1 binding) (ΔESE-1) was constructed (Figure 4D). Compared with ESE-1, the upregulatory effect on GP73 of ΔESE-1 was impaired in Hep3B and Huh7 cells (Figure 4E). These findings signified that ESE-1 overexpression increased GP73 expression.By preparing an efficient retroviral shRNA vectors for ESE-1, we also assessed whether ESE-1 upregulated GP73 expression in the presence of IL-1β. The transcript and protein levels of GP73 decreased with ESE-1 knock-down in Hep3B (Figure 4F) and Huh7 cells (Figure 4G) with or without IL-1β. GP73 protein level recovered after ESE-1 expression in both knock-down cells (Figure 4H and I). These results indicated that ESE-1 upregulated GP73 expression.

### ESE-1 bound to and activated *GP73*promoter

**Figure 5:**
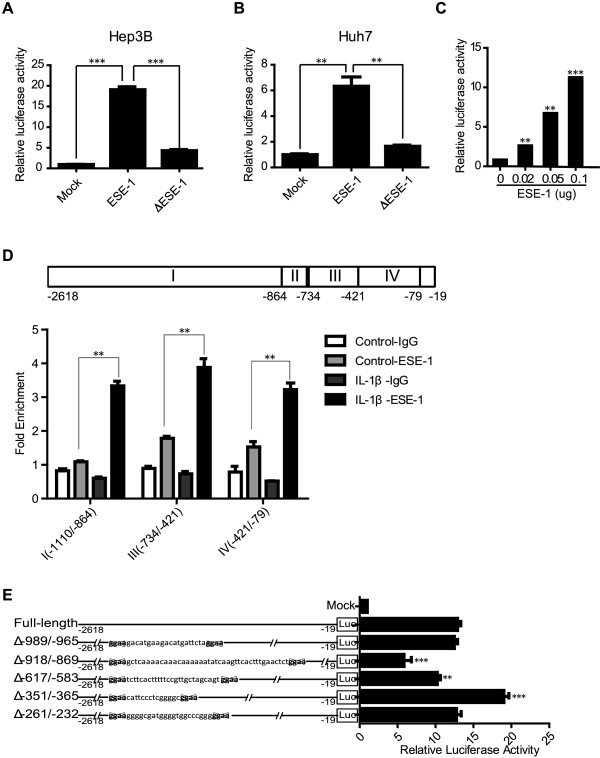
**ESE-1 bound to and activated** ***GP73*** **promoter. (A)** The empty vector (Mock) or ESE-1 or ΔESE-1 and *GP73* promoter were co-transfected into Hep3B cells. The activity of *GP73* promoter was measured by performing luciferase reporter assays after 36 h. **(B)** Similar to Hep3B cells, the activity of *GP73* promoter was analyzed in Huh7 cells as **(A)**. **(C)** Different doses of pCR3.1-ESE-1 plasmid and *GP73* promoter were co-transfected into Hep3B cells. The activity of *GP73* promoter was analyzed by completing luciferase reporter assays 36 h after transfection. **(D)** The sketch map of *GP73* promoter is shown above. The possible ESE-1 binding sites were located in regions I (−1110/–864), III (−734/–421), and IV (−421/–79) of *GP73* promoter. The results of ChIP–qPCR for ESE-1 in Hep3B cells are shown below. **(E)** The pCR3.1-ESE-1 plasmid and *GP73* full-length promoter or deletion ESE-1 binding sites mutants were co-transfected into Hep3B cells. The activity of the promoters was analyzed by accomplishing luciferase reporter assays 36 h after transfection.

To illustrate details of how ESE-1 regulated GP73 expression, ESE-1 (or ΔESE-1) and *GP73* promoter reporter vectors were co-transfected into hepatocytes, Huh7 and Hep3B. We found that ESE-1 but not ΔESE-1 efficiently activated *GP73* promoter (Figure 5A and B). Moreover, ESE-1 activated *GP73* promoter in a dose-dependent manner (Figure 5C). These results indicated that ESE-1 activated *GP73* promoter.

Based on the ESE-1 binding core sequences, the possible ESE-1 binding sites were predicted in regions I (−1110/–864), III (−734/–421), and IV (−421/–79) of *GP73* promoter (Figure 5D, upper panel). To confirm that ESE-1 directly interacted with *GP73* promoter, the specific binding of ESE-1 to these DNA sequences was analyzed with ChIP assay, followed by qPCR. Compared with the IL-1β untreated control, ESE-1 loading onto the corresponding promoters was significantly enhanced after IL-1β stimulation (Figure 5D, lower panel), indicating that ESE-1 bound to the three predicted binding sites of *GP73* promoter, and this binding activity was strongly enhanced by IL-1β treatment.

To further confirm that ESE-1 could bind to the three regions of *GP73* promoter, the corresponding deletion mutants were constructed in these regions by deleting the two adjacent possible ESE-1 binding sites. Δ–989/–965 and Δ–918/–869 deletion mutants were located in region I; Δ–617/–583 was located in region III; and Δ–351/–365 and Δ–261/–232 were located in region IV. ESE-1 and *GP73* full-length promoter (or deletion mutant promoters) were co-transfected into Hep3B cells. The results of luciferase reporter assays demonstrated that the activities of Δ–918/–869 and Δ–617/–583 mutant promoters were impaired compared with that of the *GP73* full-length promoter, whereas the activity of Δ–351/–365 was enhanced (Figure 5E). Therefore, ESE-1 could bind to regions I and III with activating effect and region IV with repressive activity. These results demonstrated that ESE-1 bound to *GP73* promoter in multiple binding sites, which was consistent with the results of the ChIP–qPCR assay.

The above findings collectively attested that ESE-1 upregulated GP73 expression in HCC cells by directly binding to and activating its promoter.

## Discussion

GP73 is elevated in many liver diseases and is considered a potential biomarker for HCC. However, little information is known about the mechanism leading to elevated GP73 expression in liver diseases. Therefore, understanding the mechanism of GP73 upregulation is of importance to develop better diagnostic and therapeutic methods. The aim of this study is to investigate the underlying mechanism of GP73 upregulation in liver diseases.

GP73 expression is up-regulated in various hepatitis and HCC, with the highest in serum of HCC patients [7]. Many studies reported that HCC can be promoted and exacerbated by inflammation [23–26]. Chronic inflammation is associated with persistent hepatic injury and regeneration, leading to sequential development of fibrosis, cirrhosis, and eventually HCC. Recently, several inflammation-related signaling pathways, such as the nuclear factor-kappa B and signal transducer and activator of transcription pathways, have been identified as key players in these processes. To reveal the regulation mechanism of GP73 expression in liver diseases, we analyzed GP73 expression in HCC cells under inflammation conditions. We found that GP73 expression was upregulated upon IL-1β stimulation *in vitro* (Figure 1) and was triggered during liver inflammation *in vivo* (Figure 2). These findings conformed to previous stipulation that GP73 is upregulated under inflammatory conditions. The regulation mechanism of GP73 expression was further realized by seeking for the key transcription factors. We discovered that ESE-1 could significantly activate *GP73* promoter (Figure 5A). Based on the fact that both ESE-1 and GP73 are expressed in epithelial cells [2, 12], and both are induced in response to IL-1β stimuli, our findings suggest that ESE-1 is at least one of the regulatory links between the inflammatory stimuli and the enhanced GP73 expression.

Since the ETS domain of ESE-1 is responsible for DNA binding, it is reasonable that the upregulatory effect on GP73 of ΔESE-1 was impaired when compared with ESE-1 in Hep3B and Huh7 cells (Figure 4E). However, recent studies showed that the ETS domain of ESE-1 also mediated the protein-protein interactions [27], and ESE-1 also contains a Pointed domain [12], an A/T hook domain [12], and a serine- and aspartic acid-rich domain [21]. Therefore, it is not surprising to find that ΔESE-1 overexpression in Hep3B and Huh7 cells showed minor discrepancy in inducing GP73 expression compared with mock (Figure 4E), which is probably related to different partners interacted with ESE-1 through different domains in different cell context [27]. Multiple ETS sites, including regions I (−1110/–864), III (−734/–421), and IV (−421/–79), were predicted on *GP73* promoter based on the ESE-1 binding core sequence. The results of the ChIP–qPCR assay (Figure 5D) and deletion ESE-1 binding site mutants (Figure 5E) confirmed that multiple ESE-1 binding sites were available in *GP73* promoter. Similar results of a regulatory region with several ETS binding sites are observed in other ESE-1 regulated genes, such as SPRR2A, Endo A [12], TGF-βII receptor [28], COX2 [15], and COL2A1 [17]. The observation of impaired activities by Δ–918/–869 and Δ–617/–583 mutants and the enhanced activity by Δ–351/–365 mutant (Figure 5E) suggested that ESE-1 could interact with either co-activators or co-repressors and may result in different responses. This postulation was supported by the fact that ESE-1 represses target gene expression by interacting with Ku70 and Ku86 proteins, but yields such expression by interacting with p300 and CREB-binding proteins [27].

In HCC, GP73 is upregulated in liver tissue and serum [5–7]. However, the role of ESE-1 in HCC has never been reported. This is the first report of the correlation between ESE-1 and HCC. Previous studies suggested that the intercellular location of ESE-1 is determined by nuclear localization and nuclear export signals [21, 22]. We found that ESE-1 located in cytoplasm and nuclear in Hep3B and Huh7 cells, regardless of IL-1β stimulation (Additional file 2: Figure S2). Nevertheless, ESE-1 was mainly distributed in the cytoplasm in liver tissue of HCC patients, which is consistent with the location of ESE-1 in human breast cancer [21, 22]. Additionally, ESE-1 and GP73 expressions were identified to be elevated and exhibited similar distribution in the liver tissue of HCC patients (Figure 3). Importantly, ESE-1 is upregulated in human cancers and prompts cancer development [18, 29, 30]. These findings were important in understanding the role of GP73 in HCC development and progression.

The results of this study indicated that ESE-1 was an important new contributing factor in controlling GP73 transcription in HCC cells. However, the delay in GP73 mRNA induction compared with ESE-1 after IL-1β stimulation *in vitro* (Additional file 3: Figure S3A and S3B) and during liver inflammation *in vivo* (Figure 2B) indicated that other factors activated after ESE-1 worked in conjunction with ESE-1 to drive GP73 transcription in response to inflammatory stimuli. A similar finding was observed when the effects of ESE-1 on matrix metalloproteinase13 transcriptional control were investigated in articular chondrocytes under proinflammatory stress [31].

## Conclusions

This study identified that ESE-1 and GP73 expressions were induced by IL-1β stimulation, and ESE-1 upregulated GP73 expression in HCC cells by directly binding to and activating its promoter. This study provided a possible mechanism for GP73 upregulation in liver diseases. More detailed investigations on the molecular mechanisms of GP73 expression is expected to contribute to understanding its functional implications in diseases and evaluating its role as a novel cancer marker.

## Methods

### Cell culture

Hep3B, HepG2, Huh7, and HEK 293T cells were maintained in a Dulbecco’s modified Eagle’s medium (Gibco, USA) supplemented with 10% fetal bovine serum (FBS) (Hyclone, USA), 100 units/mL penicillin, and 0.1% (w/v) streptomycin. For cells treated with cytokines, 10% FBS-cultured medium was removed and washed twice with PBS before adding 2% FBS-cultured medium with IL-1β (Peprotech, USA).

### Plasmid construction and virus preparation

Flag epitope-tagged ESE-1 from the pCI-ESE-1 plasmid [12] was subcloned into pCR3.1 plasmid with Hind III and EcoR V to produce pCR3.1-ESE-1 plasmid. pCR3.1-Δ ESE-1 was generated by removing the ETS domain (272 to 354 amino acids) of ESE-1 [12]. Meanwhile, deletion ESE-1 binding site mutant promoters were formed by fusing polymerase chain reaction (PCR) based on the PGL3-basic firefly luciferase reporter vector with *GP73* promoter (−2618/−19) [32]. All PCR primers are shown in Additional file 4: Table S1.

Plasmid pSuper-shESE-1, which was used to generate siRNA against ESE-1, was constructed by inserting the target sequence into pSuper (Oligoengine, USA) in site with Bgl II and Hind III. The inserted sequences are specified in Additional file 4: Table S1. Infectious shESE-1 virus particles were then generated by co-transfecting HEK293T cells with pSuper-shESE-1 and packaging plasmid [33, 34]. At 48 h after transfection, the supernatant that contained the virus was filtered through 0.45 μm filters and was incubated with the target cells with 4 mg/mL polybrene (Sigma, USA).

### Quantitative PCR

For mRNA quantification, total RNA from cells or liver tissue was prepared with Trizol reagent according to the manufacturer’s protocol (Sangon). cDNA was synthesized using a reverse transcription kit (Takara). SYBR real-time PCR was performed with specific primers (Additional file 4: Table S1) in a CFX96 Real-Time PCR Detection System (Bio-rad, USA), and glyceraldehyde 3-phosphate dehydrogenase (GAPDH) or 18S was used as an internal control for standardization. All reactions were performed in triplicate.

### Western blot analysis

Cells were resuspended in 100 μL of radio immunoprecipitation assay (RIPA) buffer (Beyotime) supplemented with protease inhibitor cocktail (Roche). Cell lysate was analyzed by using 12% SDS-PAGE and was transferred onto polyvinylidene fluoride membranes. 5B12 [7] and ab1392 (Abcam) antibodies were applied to detect GP73 and ESE-1 proteins, respectively. Protein level was normalized by GAPDH, a housekeeping protein detected by mouse anti-GAPDH monoclonal antibody (Kang Xiang, Shanghai).

### Transfection and reporter assays

Cells (2 × 10^4^) were seeded in 96-well culture plates 12 h before transfection. The GP73 promoter and pCR3.1-ESE-1 plasmid or Δ ESE-1 or empty vectors together with pRL-TK plasmids were cotransfected into the cells with Lipo2000™ (Invitrogen). The cells were harvested 36 h after transfection and were analyzed by using the Dual-glo luciferase assay kit (Promega). All transfections were performed in triplicate and repeated at least thrice. Firefly luciferase activity was normalized to renilla luciferase activity for each sample.

### Chromatin immunoprecipitation assay

Chromatin immunoprecipitation (ChIP) assays were performed by using ChIP assay kit (P2078, Beyotime) following the manufacturers’ instructions. Hep3B cells (1 × 10^7^) were plated and transfected with Flag-tagged ESE-1 expression vectors and were subsequently incubated with or without 10 ng/mL IL-1β for 2 h. After 36 h, cross-linking was performed with 1% formaldehyde for 10 min at room temperature. Nuclei membrane was impaired, and chromatin was sheared by sonication for 7 min to 10 min, producing 250 bp to 1000 bp fragments. Chromatin was then incubated overnight after mixing with 50 ul of protein G/A magnetic beads (Life Technology) and 4 ul of nonspecific mouse IgG (Chemicon) or 4 ul of monoclonal mouse Flag antibody (F1804, Sigma) for 6 h at 4°C with rotation. DNA was precipitated using 10% Chelex-100, which was subjected to quantitative PCR (qPCR) analysis. The primers used are listed in Additional file 4: Table S1. Negative control was performed by using the control primer of the kit.

### Immunohistochemical and immunofluorescence analysis

The mouse liver inflammation model was constructed by intraperitoneally injecting 35 ng/kg of LPS and 250 mg/kg of D-galactosamine [19]. The protocol was approved by the Institutional Animal Care and Use Committee of the Guangzhou Institutes of Biomedicine and Health, Chinese Academy of Sciences (IACUC ID: 2013016). Liver tissues were collected at different time points and were subsequently fixed and sectioned (3 μm thick). By using antibody 10B4 prepared in the laboratory (Additional file 5: Figure S4) and anti-mouse HRP-conjugated secondary antibody, mouse GP73 was detected and subsequently stained with 3,3′-fiaminobenzidine solution (ZSGB-BIO, Beijing). The sections were visualized with motic digital slices scanning and application system (Motic VM V1).

For immunofluorescence analysis, full-thickness human liver tissue samples were obtained from the Guangzhou Overseas Chinese Hospital. These tissue samples were fixed and sectioned (3 μm to 4 μm thick), and hematoxylin–eosin (HE) staining was completed in successional sections. Human GP73 was localized using antibody 5B12 and was incubated with sheep anti-mouse immunoglobulin conjugated to fluorescein isothiocyanate (Chemicon, USA). ESE-1 was labeled using ab1392 antibody (Abcam) and was incubated with goat anti-rabbit antibody conjugated to rhodamine (Chemicon, USA). Nuclei were counterstained with 4,6-diamidino-2-phenylindole (DAPI; KPL, USA). The sections were viewed with a confocal microscope (LSM 710; Zeiss, Germany).

### Statistical analysis

Data were reported as mean ± standard error of at least three independent experiments, and statistical analysis was performed by using ANOVA, followed by Student’s t-test (*: p < 0.05; **: p < 0.01; ***: p < 0.001).

## Electronic supplementary material

Additional file 1: Figure S1: The correlation of ESE-1 and GP73 expression in other hepatocytes. Human hepatocytes: Bel-7402, QSG-7701, HL-7702(L-02); Mouse hepatocytes: Hep1-6, AML-12. (PDF 254 KB)

Additional file 2: Figure S2: The location of ESE-1 protein in Hep3B and Huh7 cells with or without IL-1β stimulation. (PDF 588 KB)

Additional file 3: Figure S3: The time course expression of ESE-1 and GP73 mRNA in Hep3B cells with IL-1β stimulation. (PDF 313 KB)

Additional file 4: Table S1: Sequences of PCR primers used in this study. (DOCX 15 KB)

Additional file 5: Figure S4: Validation of mouse GP73 (10B4) antibody. (A) Flag epitope-tagged pCR3.1-mGP73 plasmids were transfected into 293T cells; mouse GP73 (mGP73) expression was detected using anti-flag antibody (F1804, Sigma) or anti-mGP73 antibody (10B4) with Western blot. (B) The location of mGP73 was tested using 10B4 antibody in Hep1-6 cells with immunocytochemistry. Black arrows signified the location of mouse GP73 protein. (PDF 1 MB)
