# Supplementary figures and images for: Epithelium-Specific ETS (ESE)-1 upregulated GP73 expression in hepatocellular carcinoma cells
Source: Cell Biosci. 2014 Dec 8;4:76. doi: 10.1186/2045-3701-4-76 (PMC4271417; doi:10.1186/2045-3701-4-76)

Figure S1

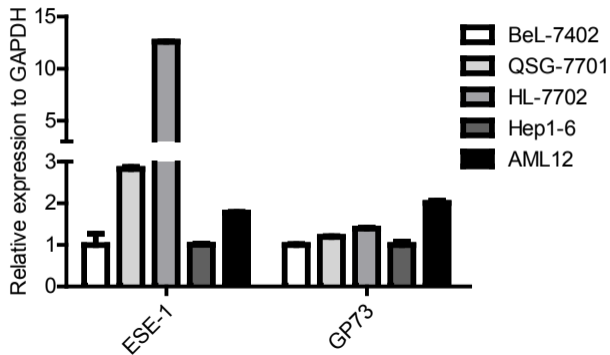

Supplement: Supplementary file 1 — Additional file 1: Figure S1: The correlation of ESE-1 and GP73 expression in other hepatocytes. Human hepatocytes: Bel-7402, QSG-7701, HL-7702(L-02); Mouse hepatocytes: Hep1-6, AML-12. (PDF 254 KB) [file 13578_2014_197_MOESM1_ESM.pdf]

Figure S2

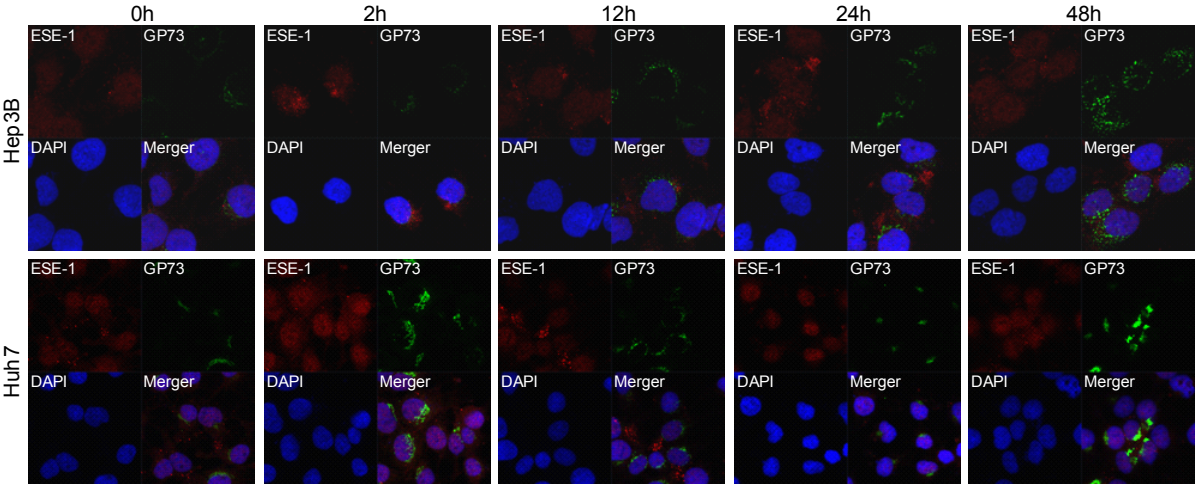

Supplement: Supplementary file 2 — Additional file 2: Figure S2: The location of ESE-1 protein in Hep3B and Huh7 cells with or without IL-1β stimulation. (PDF 588 KB) [file 13578_2014_197_MOESM2_ESM.pdf]

Figure S3

**A**

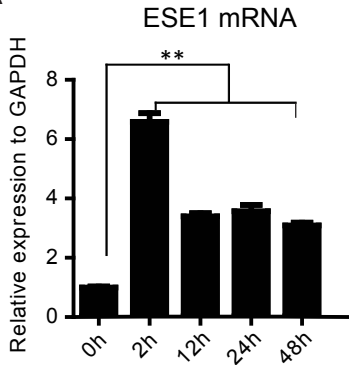

**B**

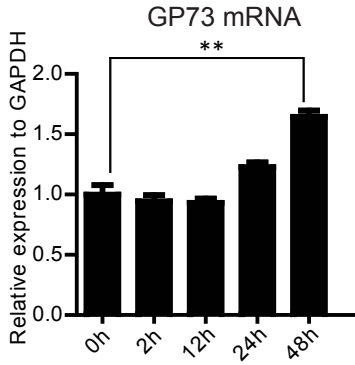

Supplement: Supplementary file 3 — Additional file 3: Figure S3: The time course expression of ESE-1 and GP73 mRNA in Hep3B cells with IL-1β stimulation. (PDF 313 KB) [file 13578_2014_197_MOESM3_ESM.pdf]

Figure S4

A

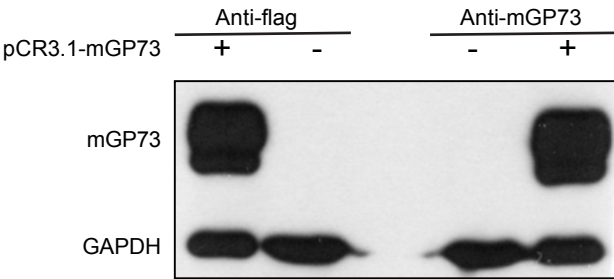

B

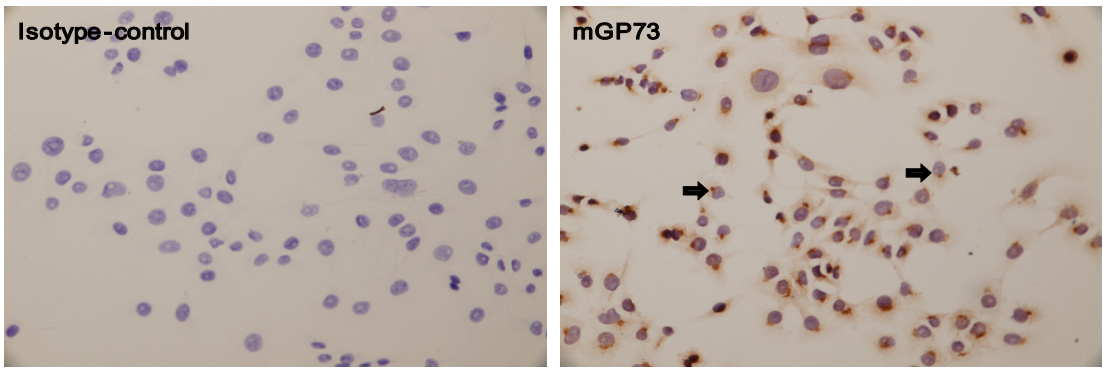

Supplement: Supplementary file 5 — Additional file 5: Figure S4: Validation of mouse GP73 (10B4) antibody. (A) Flag epitope-tagged pCR3.1-mGP73 plasmids were transfected into 293T cells; mouse GP73 (mGP73) expression was detected using anti-flag antibody (F1804, Sigma) or anti-mGP73 antibody (10B4) with Western blot. (B) The location of mGP73 was tested using 10B4 antibody in Hep1-6 cells with immunocytochemistry. Black arrows signified the location of mouse GP73 protein. (PDF 1 MB) [file 13578_2014_197_MOESM5_ESM.pdf]
