# Supplementary material for: Epithelium-Specific ETS (ESE)-1 upregulated GP73 expression in hepatocellular carcinoma cells
Source: Cell Biosci. 2014 Dec 8;4:76. doi: 10.1186/2045-3701-4-76 (PMC4271417; doi:10.1186/2045-3701-4-76)
Supplement: Supplementary file 4 — Additional file 4: Table S1: Sequences of PCR primers used in this study. (DOCX 15 KB) [file 13578_2014_197_MOESM4_ESM.docx]

**Additional file 1: Table S1 Sequences of PCR primers used in this study**

| Contents | Primer sequences |
| --- | --- |
| qPCR primers | h^a^GP73-F: 5’ TGGCAGGGAATGACAGAAA3’  hGP73-R: 5’ TCGGCCCTGTTGTGAAAT3’  hGAPDH-F: 5’ TTGCCATCAATGACCCCT3’  hGAPDH-R: 5’ CATCGCCCCACTTGATTT3’  hESE-1-F: 5’GTGGAAGTGACGTGGACCT3’  hESE-1-R: 5’TTCCGCTTCCCGTGCTT3’  m^b^GP73-F: 5’CCCAGGAGATGAATACGACATG3’  mGP73-R: 5’GTCCATTTCACAGTTGAGTCAC3’  mESE-1-F: 5’GGCCCTCATGGCTGCCACCT3’  mESE-1-R: 5’TTGGGATCTTGTCTGAGGTCCTGGA3’  18S-F: 5’GGTGAAATTCTTGGACCGGC3’  18S-R: 5’GACTTTGGTTTCCCGGAAGC3’ |
| shESE-1 | 5’GATCCCCACAGCAACATGACCTACGATTCAAGAGATCGTAGGTCATGTTGCTGTTTTTTA3’  3’GGGTGTCGTTGTACTGGATGCTAAGTTCTCTAGCATCCAGTACAACGACAAAAAATTCGA5’ |
| ΔESE-1 | Sca1-F:5’ ACTGCAAGAAGGGGGATCCCAAGCAC3’  deETS-fusion-R:5’GCTTGAGTTTTTGCCTCTGGGCGCGTGCTTGCTCTTCTTG3’  deETS-fusion-F:5’AAGCACGCGCCCAGAGGCAAAAACTCAAGCGGCTGGAAGG3’  Xho1-R: 5’ATACTCGAGCGGCCGCCACTGTGCTGGA3’ |
| Deletion ESE-1 binding sites mutants | Pst1-F: 5’TGTCTGCAGTTAAGAAGCTCCATGCTCAG3’  Xhol-R: 5’ATACTCGAGCTCCGGCCGGCTCCGCGCCGTGCGCC3’  Δ-989/-965-F: 5’ AACTGTTCTCAATCTTGGCCGTATA3’  Δ-989/-965-R:5’TATACGGCCAAGATTGAGAACAGTTTAGAATCATGTCTTCATGTCTAGCTCTGGTGTCTGGGAAAGGAAA3’  Δ-918/-869-F: 5’ GTGGGACCCTGGTTCCTAAATGCTC3’  Δ-918/-869-R:5’GAGCATTTAGGAACCAGGGTCCCACAGAGTTCAAAGTGAACTTGATATTTTTTGTTTGTTTTGAGCCAGATAATCCTAATACAGTATACGG3’  Δ-617/-583-R:5’GGCCTTAGTGTTTGGTCTGTGACCCACTGCTAGCAACGGAAAAAGTGAAGACGGACGCCTTCGGGTGTGATCTTAT3’  Δ-617/-583-F: 5’ GGGTCACAGACCAAACACTAAGGCC3’  Δ-351/-365-R:5’ACCTGGGGGGAGCCCCCATCCCACGGCCCCGAGGGAATGCGAACAGCCAACCGCACCCAGCGCC3’  Δ-351/-365-F: 5’ CGTGGGATGGGGGCTCCCCCCAGGT3’  Δ-261/-232-R:5’AGTTCTCGCCGCTGGGCGTTGCCCCCCCGGGCCACCCCATCGCCCCGACGCGGGAGCTGGCGCCTGAGCAG3’  Δ-261/-232-F: 5’ GGGGCAACGCCCAGCGGCGAGAACT3’ |
| ChIP-qPCR | Ⅰ(-1110/-864)-F: 5’TGATTTGAGAGAGGCGCA3’  Ⅰ(-1110/-864)-R: 5’CGGCCAAGATTGAGAACAG3’  Ⅲ(-734/-421)-F: 5’AGGCGCTGTTTCAAATGC3’  Ⅲ(-734/-421)-R: 5’TTGGTCTGTGACCCTTCCA3’  Ⅳ(-421/-79)-F: 5’GTAACGTGAGTGTGGCGCT3’  Ⅳ(-421/-79)-R: 5’TGGGGAAAGGGTACCTGG3’ |

a: h, human;

b: m, mouse;
